# Noggin Nodding: Head Movement Correlates With Increased Effort in Accelerating Speech Production Tasks

**DOI:** 10.3389/fpsyg.2019.02459

**Published:** 2019-11-27

**Authors:** Mark Tiede, Christine Mooshammer, Louis Goldstein

**Affiliations:** ^1^Haskins Laboratories, New Haven, CT, United States; ^2^Institut für Deutsche Sprache und Linguistik, Humboldt-Universität zu Berlin, Berlin, Germany; ^3^Department of Linguistics, University of Southern California, Los Angeles, CA, United States

**Keywords:** speech production, speech errors, head movement, EMA, articulatory entrainment

## Abstract

Movements of the head and speech articulators have been observed in tandem during an alternating word pair production task driven by an accelerating rate metronome. Word pairs contrasted either onset or coda dissimilarity with same word controls. Results show that as production effort increased, so did speaker head nodding, and that nodding increased abruptly following errors. More errors occurred under faster production rates, and in coda rather than onset alternations. The greatest entrainment between head and articulators was observed at the fastest rate under coda alternation. Neither jaw coupling nor imposed prosodic stress was observed to be a primary driver of head movement. In alternating pairs, nodding frequency tracked the slower alternation rate rather than the syllable rate, interpreted as recruitment of additional degrees of freedom to stabilize the alternation pattern under increasing production rate pressure.

## Introduction

Movements of the head are integral to human speech. Casual observation of any conversational interaction reveals head nodding employed by the current speaker aligned with prosodic features and by listeners providing backchannel feedback. Nodding is coordinated with and complementary to other forms of gesticulation like hand and facial movements (Wagner et al., 2014), and sensitive to speech rate and affect (Birdwhistell, 1970; Giannakakis et al., 2018). Head movements are used by speakers to structure discourse (Kendon, 1972), indicate deixis (Birdwhistell, 1970; McClave, 2000) flag lexical repair (McClave, 2000), and to signal a turn-taking shift (Duncan, 1972; Hadar et al., 1984a), among other functions.

However, these ***communicative*** uses of head movement also coexist with ***motoric*** consequences of speech production, like those to be discussed in this work. These include head adjustments for respiration or compensations for other body movement (e.g., talking while walking; Raffegeau et al., 2018) as well as head movement entrained through the influence of active articulation. For example, in a kinematic study of head movement during conversation, Hadar et al. (1983b, p. 40) observed that during speaking turns, “the head moved almost incessantly”, with 89.9% of recorded frames showing non-zero velocity. This contrasted with relatively little movement during pauses and listening turns (12.8%). In a follow-on analysis, they found a significant positive correlation between head movement amplitude and peak speech loudness (Hadar et al., 1983a) but observed that this was driven mostly by fast, high-intensity movements and loud sounds. Similarly, a study investigating emotionally contrastive speech tasks (elicited using neutral *vs.* psychologically stressful interviews) found significantly increased head velocity under the stressed condition, corroborated by increases in concurrently recorded heart rate (Giannakakis et al., 2018). Congenitally blind speakers have been shown to move their heads while speaking with non-sighted speech partners, showing that speech entrains head movement despite, in this instance, lacking a communicative role that would usually be expressed through the visual channel (Sharkey and Stafford, 1990). Another relevant study by Hadar (1991) measured the head movement of aphasics and normal controls engaged in speech during interviews. He found that while head movement was positively correlated with speaking rate for both groups, it was highest for non-fluent aphasic speakers who, apart from increased effort required for speech coordination, showed no other motor impairment.

Yehia et al. (2002) used point source (Optotrak) data collected for sentence productions of two speakers to estimate F0 from head motion and vice versa. Their results showed 88 and 73% of F0 variance accounted for by head motion for the two speakers, respectively, but just 50 and 25% of head motion variance accounted for by F0 in the reverse direction. This asymmetry is consistent with the likelihood that competing demands on head position imposed by communicative intent distort estimates driven by prosodic F0 alone, but it leaves open the question of why, in the opposite direction, head movement should be so effective at predicting F0. Following Honda (2000), they suggest that strap muscles connecting the floor of the mouth through the hyoid bone and attaching to the outer edge of the cricothyroid cartilage provide an indirect biomechanical coupling, such that as the head is tilted, the straps will exert pull on the cricothyroid and thus potentially influence vocal fold tension. Although any such effect would be small, it might nonetheless serve to entrain modulation between head movement and F0.

A similar pattern of loose coupling is illustrated by a non-speech task in which Kohno et al. (2001) asked four participants to open and close their mouths, tapping their teeth together in the closing cycle, while tracking movement of the upper and lower incisors. Jaw opening ranges were 1, 2, and 3 cm, and tapping frequency elicited by metronome varied from 1 to 3.3 Hz. Except for the smallest and slowest condition, the upper incisor was observed to move up at the same time that the lower incisor moved down at about 10% of its range. Cycle durations for both were found to be highly correlated (*r* = 0.94) and so were their vertical ranges of movement (*r* = 0.75). They propose that this coordination of movement may serve to make jaw movements smoother through offsetting postural changes of the head. While this likely occurs primarily during mastication, it suggests that rhythmic movement of the jaw during speech may also entrain head movement.

However, while it appears that motoric aspects of speech production can and often do affect head movement, such influence is neither automatic nor readily predictable. For example, Rimé et al. (1984) and Hoetjes et al. (2014) contrasted conversational speech in a baseline condition when speakers were free to move with a condition in which the head and other extremities were immobilized and reported no difference in speech fluency; this makes clear the lack of any direct biomechanical linkage between the speech articulators and the head. What then is the cause of non-communicative head movement linked to speech? One possibility is that the head participates somehow in networks of “coordinative structures” assembled as needed to achieve particular motor goals (Kugler et al., 1980) while constraining the degrees of freedom under control (Bernstein’s Problem; Bernstein, 1967). Such structures, provided with appropriate input energy, dissipate it in a controlled and stable fashion, provided that the control parameters themselves are consistent; however, if these change beyond some threshold, driven say by execution errors or an increase in production rate, additional degrees of freedom are recruited as a new structure is organized (Kelso et al., 1993). Two studies from Dittmann and Llewellyn (1969) and Hadar et al. (1984b) are suggestive in this context: they report that the amplitude of head movement increases spontaneously immediately following speech dysfluencies. In this case, movement of the head appears to be recruited to serve a phase-resetting function for the interrupted articulatory plan by introducing additional energy and stability into the coordinative structures executing it (e.g., Fowler et al., 1980; Saltzman and Munhall, 1989). Because head movement does not contribute directly to achievement of the articulatory target, the linkage between the head and the articulatory system is a functional one, introduced by extending the coordinative structure to include the head as necessary.

The sensitivity of head movement to speech dysfluencies suggests that a useful paradigm for studying its relationship to articulation is through a task designed to elicit such errors reliably. Previous work has established that the repetition of word pairs with partial similarity (e.g., *top cop*) results in more production errors than either identical or entirely dissimilar words (Meyer and Gordon, 1985), and that alternating codas are slower to produce and more errorful than alternating onsets (Sevald and Dell, 1994). Kinematic studies of such sequences have confirmed this asymmetry (Mooshammer et al., 2018) and have shown that systematic alternation can lead to inappropriate suppression of the target constriction (a reduction error) or co-constriction of the non-targeted articulator (an intrusion), which in both cases may be partial or subphonemic (Pouplier, 2003; Goldstein et al., 2007). Kinematic studies of alternating sequences have also shown that more errors occur at higher production rates and that intrusion errors are more common (Goldstein et al., 2007; Slis and Van Lieshout, 2016).

An explanation for this behavior advanced in Goldstein et al. (2007) rests on the idea that during repetition, the executing task becomes a system in which each constriction *gesture* (lips, tongue tip, and tongue body) is driven by a non-linear oscillator, and those oscillators are coupled through synergy with the shared jaw. However, the frequencies of all of the oscillators are not the same because of the mismatch between the syllable rate *vs.* the alternating (phrasal) rate. In *top cop*, for example, the alternating tongue tip and tongue dorsum constrictions occur at one half the rate of the bilabial closures, and this 1:2 frequency ratio is inherently less stable than a 1:1 relationship. It is known from studies of coupling between non-linear oscillators that their mutual phasing preferentially shifts from less stable to more stable patterns of organization, with the simplest 1:1 mode ultimately preferred (Haken et al., 1985). In addition, a series of index finger-wagging experiments has demonstrated that as rate increases, the end result, regardless of starting conditions, is in-phase symmetric motion at the 1:1 rate (e.g., Kelso et al., 1993). Speech errors of the co-constriction type can thus be viewed as incipient phase transitions, which may either be transitory, if the production system succeeds in resetting itself, or complete. The expected effect of recruiting an additional oscillator such as the head at the lower frequency (phrasal) rate would be to bias the system to remain in the 1:2 mode: the idea is that the more power is shared among the oscillating components at a given frequency, the more stable that frequency will be (Nam et al., 2009).

An alternative view arises from kinematic studies of constriction variability (interpreted as gradient production errors) in repeated word pairs with alternating onsets conducted by Slis and Van Lieshout (2013, 2016). They report higher rates of tongue dorsum instrusion in onset alternation, especially in high (constrained) vowel contexts, relative to lower intrusion rates for tongue tip and lower lip constrictions, and more intrusions than reductions overall. They attribute this to potential co-production demands on the primary constriction articulator, which can serve to bias a shared articulator toward partial or complete co-constriction as a consequence of coupling dynamics between gestures. In this view, the *fewer* shared oscillatory components (articulators) utilized to achieve an articulatory target, the less susceptible it will be to such bias. Thus, because the lower lip apart from the jaw is uncoupled from the tongue, it “is better able to maintain linguistic goals and counteract pressure from coupling forces to stabilize coordination patterns” (Slis and Van Lieshout, 2016, p. 14).

Irrespective of their cause, it is clear that the alternating word paradigm reliably produces errors and has, in the context of this current work, the additional advantage of minimizing communicative gesturing of the head (given the rote nature of the task), such that observed head movement can for the most part be attributed to motoric consequences of articulating the sequence (although a possible exception, the use of head movement to emphasize phrasal stress, will be explored below). Accordingly, this work uses the alternating word paradigm to investigate relationships between head movement and speech articulation. It extends previous work in two ways. First, production of alternating word pairs is driven by an accelerating rather than fixed rate metronome. This has the advantage of contrasting an initial low stress production rate (with a constant metronome period) against the effects of subsequent rate acceleration, placing the speaker under increasing production effort, with errors increasingly likely. Second, the motion of the head is tracked in tandem with observation of the speech articulators to investigate the effects of increasing production rate and effort on the following research questions:

• Does increased production rate correlate with increased head movement?
• Is head movement sensitive to onset *vs.* coda asymmetry?
• Does head movement increase following production errors? Is this dependent upon error type?
• Is increased head movement a function of increased jaw movement?
• Is head movement driven by an imposed prosodic stress pattern?

With the consideration that recruitment of the head, if it occurs, is expected to support 1:2 alternation, we also evaluate the following hypothesis:

***H*_1_**: In the production of alternating word pairs, the moving head will track the slower (phrasal) rate rather than the syllable rate frequency.

The approach to addressing these questions is outlined below.

## Materials and Methods

### Participants

Nine native speakers of American English (five females, mean age 24.4) were recruited from the New Haven community for the experiment. None reported any neurological, speech, or hearing disorders. Each provided informed consent supervised by the Yale University Institutional Review Board and were paid for their participation.

### Recordings

Speech articulator movements with synchronous audio were recorded using electromagnetic articulometry (EMA; Carstens AG500). For each participant, EMA sensors were affixed using dental cyanoacrylate to the tongue dorsum (TD), blade (TB), and tip (TT), the upper (UL) and lower (LL) lips, and lower incisors (JAW) along the midsagittal plane. The TD sensor was placed as far back as the participant could comfortably tolerate; the TT sensor was placed approximately 1 cm posterior to the apex; and the TB sensor was centered between these. Lip sensors were attached at the vermillion border, and sensors placed on the upper incisors (UI) and JAW were attached at the gingival margin. Additional sensors placed on the left and right mastoid processes and nasion were used as references to correct for head movement. Biteplane data were collected to establish the occlusal plane for each participant. Three spatial dimensions for position were sampled for each EMA sensor at 200 Hz. Synchronized audio was recorded with a 16-kHz sampling rate using a directional microphone placed approximately 50 cm from the participant’s mouth. Metronome clicks used to pace production as discussed below were presented monaurally through an earpiece in the left ear (opposite from EMA wires) and recorded separately at 8 kHz.

### Speech Tasks

The speech material discussed here consisted of repeated CVC real English word pairs that alternated in one of three ***context*** types. In the first context (SAME), both words were identical (e.g., *top top*). In the second context (ONSET), the onset consonant of each word alternated (e.g., ***t**op **c**op*). In the third context (CODA), the coda consonant of each word alternated (e.g., *to**p** to**ck***). An additional condition in which both onset and coda were varied (e.g., *pop tot*) was also collected but is excluded from this analysis as it produced an excessive number of production errors that were not amenable to the split-mean analysis described below. Both vowels from each word pair were always the same. Note that this procedure, which elicits repetitions of the same word pair throughout a trial, differs from paradigms in which different word pairs are contrasted to facilitate spoonerisms (e.g., Nooteboom, 2005). The list of words used is given in Table 1, which were presented in a total of 39 different pairings (including reverse orderings when not identical), although not all participants produced every combination. The word pair alternation trials were collected as blocks within a larger experiment probing speech errors in production, presented in Mooshammer et al. (2018).

**TABLE 1:** Speech material.

**Context**	**Articulators**	**Sensors**	**C**	**V**	**Word Pair**	**# trials**
Same	lab/cor	LA/TT	p – d	/a/	pod pod	27
	lab/dor	LA/TD	p – k	/æ*/*	pack pack	24
	lab/dor	LA/TD	p – g	/a/	pog pog	9
	cor/lab	TT/LA	t – p	/a/	top top	27
	cor/lab	TT/LA	t – p	*/*eI/	tape tape	30
	cor/dor	TT/TD	t – k	/a/	tock tock	9
	cor/dor	TT/TD	t – k	/æ*/*	tack tack	30
	dor/lab	TD/LA	k – p	*/*eI/	cape cape	24
	dor/lab	TD/LA	k – p	/a/	cop cop	27
	dor/lab	TD/LA	k – b	/a/	cob cob	9
	dor/cor	TD/TT	k – d	/a/	cod cod	24
Onset	lab/cor, dor/cor	LA/TT, TD/TT	p – t, k – t	/i/	pit kit	45
	lab/cor, dor/cor	LA/TT, TD/TT	p – d, k – d	/a/	pod cod	51
	lab/dor, cor/dor	LA/TD, TT/TD	p – k, t – k	/æ*/*	pack tack	42
	lab/dor, cor/dor	LA/TD, TT/TD	p – k, t – k	/a/	pock tock	51
	cor/lab, dor/lab	TT/LA, TD/LA	t – p, k – p	*/*eI/	tape cape	42
	cor/lab, dor/lab	TT/LA, TD/LA	t – p, k – p	/a/	top cop	54
Coda	lab/cor, lab/dor	LA/TT, LA/TD	p – t, p – k	/æ*/*	pat pack	39
	lab/cor, lab/dor	LA/TT, LA/TD	p – d, p – g	/a/	pod pog	36
	cor/lab, cor/dor	TT/LA, TT/TD	t – p, t – k	/i/	tip tick	48
	cor/lab, cor/dor	TT/LA, TT/TD	t – p, t – k	*/*eI/	tape take	78
	cor/lab, cor/dor	TT/LA, TT/TD	t – p, t – k	/a/	top tock	45
	dor/lab, dor/cor	TD/LA, TD/TT	k – p, k – t	*/*eI/	cape Kate	39
	dor/lab, dor/cor	TD/LA, TD/TT	k – p, k – t	/a/	cop cot	51
	dor/lab, dor/cor	TD/LA, TD/TT	k – b, k – d	/a/	cob cod	45

*Trial counts are across all participants, and for alternating pairs include the reverse orderings.*

### Procedure

Trials were cued using a computer monitor that presented the instructions “Get ready – Breathe – GO” at 1-s intervals, together with the word pair under test. During the “Breathe” instruction, metronome clicking was initiated, delivered to the participant through an earpiece to avoid contaminating his or her audio production. Participants were instructed to time the onset of each word to a click and to avoid breathing during the trial if possible due to the phase-resetting effects of respiration (Goldstein et al., 2007). Some speakers were explicitly asked to produce trochaic stress while others were uninstructed for stress placement; however, all were consistent in stress realization. Metronome timing was computer-controlled to produce clicks over a 15-s interval, chosen to be readily achievable for participants to produce the entire alternation sequence within one breath. Clicks were exponentially decaying transients with a half-power bandwidth of 2 ms. During the first 7.5 s, the click rate was held stable at 170 clicks/min, following which the rate was increased with each click by a constant percentage of the current rate (0.12) to approximately 235 clicks/min at the final (48th) click. The advantage of this approach is that the initial stable rate provides an easy-to-maintain baseline for all speakers, with few production errors, while the subsequent rate acceleration places all speakers under increasing production effort, with errors increasingly likely.

### Post-processing

#### EMA Data

EMA sensor trajectories were processed in MATLAB (Mathworks) using zero-phase delay low-pass filtering at 20 Hz. The smoothed reference trajectories (UI, nasion, mastoids) were then used to rotate and translate all data to a coordinate system aligned with each speaker’s occlusal plane centered on UI, as determined by their reference position in the biteplane trial. A copy of the UI sensor trajectory (HEAD), filtered but without head correction, was used to characterize speaker head movement for each trial. Velar and alveolar closures were tracked using the TD and TT trajectories, respectively. For bilabial closures, a derived measure of lip aperture (LA) was computed as the Euclidean distance between the UL and LL sensors (In one instance, where UL data were unusable, the vertical component of LL was used instead).

#### Defining Epochs

**FIGURE 1:**
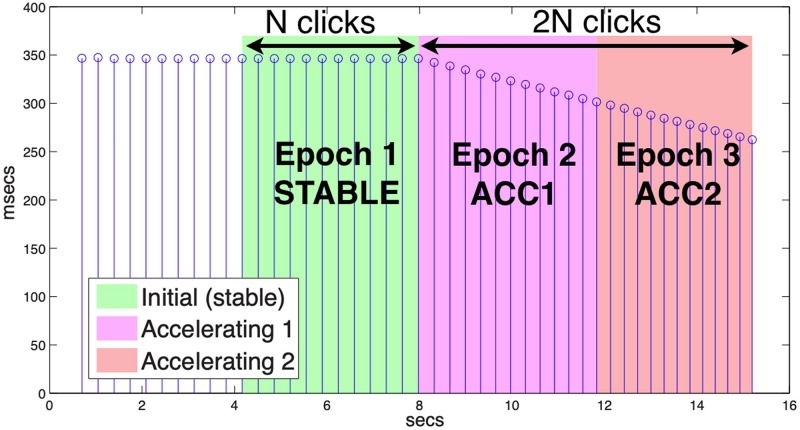
Metronome click periods. Epochs are delimited by the number of clicks between the start of acceleration and the last click with produced speech (2*n*), with each epoch including *n* clicks.

To distinguish the stable and accelerating phases of each trial, a functional grouping into epochs was determined procedurally as follows. First, the offset of each metronome click was identified by peak-picking RMS peaks within its audio channel. Next, the inflection point at which rate began to increase was found by differencing the click periods. The final usable click for a given trial was determined by inspection as the last click for which the speaker produced a controlled utterance timed to the metronome. The number of clicks from the inflection point to the final click was taken to be twice the epoch length for the trial (2*n*), such that the initial (STABLE) epoch encompassed *n* clicks preceding the inflection, the first accelerating epoch (ACC1) was *n* clicks following that, and the final accelerating epoch (ACC2) covered the remaining *n* clicks (see Figure 1 for an illustration). The minimum epoch length (*n*) was nine clicks with mean 11.6 and s.d. 2.1. Because participants always began speaking before the beginning of the STABLE epoch, and continued production until at least the final click, this method ensured that movement during each trial could be binned systematically.

#### Identifying Errors

**FIGURE 2:**
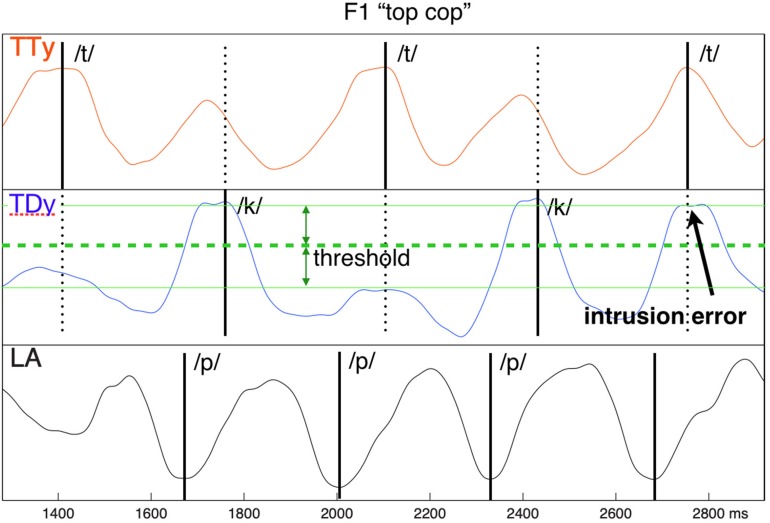
Intrusive error example, showing inappropriate co-constriction of tongue dorsum (TD) coincident with the /t/ target TT closure. The error threshold is determined as the “split” mean between the median distributions of in-phase and anti-phase articulator extrema.

Production errors were identified on the EMA trajectories using the “split-mean” criterion established by Pouplier (2008). This approach relies on establishing the distributions of in-phase and anti-phase constriction events for non-errorful productions, then using the mean between them as a threshold to identify inappropriate deviations from expected behavior. For example, in the *top cop* sequence, the upward movement of the tongue tip during the tongue constriction we will refer to as “in-phase,” while its upward movement at the time where the tongue dorsum (with which it alternates) is forming a constriction we will refer to as “anti-phase.” When the vertical component of TT fails to rise above threshold for its in-phase position (i.e., its expected target constriction), a ***reduction*** error is identified. Conversely, when it rises above threshold at its non-target anti-phase position (i.e., coincident with the expected velar constriction), an ***intrusion*** error is identified. When a reduction or intrusion error in one alternating articulator co-occurs with an error of the opposite polarity in its partner, a ***substitution*** error is identified. Following this approach, described more fully as the “error rate” procedure in Mooshammer et al. (2018), errors of these three types were labeled using a semiautomatic interactive procedure on the TD, TT, and LA trajectories of each trial. Figure 2 provides an example.

### Measurements

To investigate overall effects of increasing production rate on head movement, one set of measures was organized to contrast global effects over the three epoch phases (STABLE, ACC1, and ACC2). A separate set of measures was used to investigate local effects of errors, contrasting the immediate environment preceding and following each error (PRE, POST). Except as noted below, all measurements were computed using standard MATLAB augmented by locally developed procedures.

#### Epoch-Based Measures

Head movement was quantified over epochs on the HEAD (filtered UI) trajectory in two ways. Overall movement (MVT) was measured as the path integral distance traced by the UI sensor during each epoch, normalized by the duration of the epoch. Peak tangential velocity (VEL) was measured by first computing HEAD speed using central differencing, then computing the maximum of this signal over each click interval normalized by the duration of that interval, and finally recording the maximum of these values achieved within each epoch as the characterizing value for that epoch. In both cases, the time normalization was used to offset the effect of increasing metronome rate.

To investigate the relationships between movement of the head, the jaw, and the active articulators, we computed measures of average mutual information (AMI) and mutual power (MP). As these require comparing monodimensional signals, we used the first principal component of the HEAD and JAW trajectories and that of the alternating and non-alternating articulators as characterized by TD, TT, and LA (LA was used directly without principal component decomposition).

##### Average Mutual Information

Mutual Information (MI) quantifies the information dependency of two random variables, such that knowledge available for one reduces uncertainty associated with the other (e.g., Cover and Thomas, 2006). That is, *MI*_*ij*_ is the amount communicated by a given measurement *y*_*j*_ from *Y* about the value *x*_*i*_ measured from *X*. When this dependence is averaged over all cells in the joint distribution between *X* and *Y*, the result is their *average mutual information* (AMI), expressed in bits. In contrast to correlation, which tests only linear dependency, AMI is sensitive to the entire form of the joint distribution and thus evaluates nonlinear dependency. An AMI of zero implies that two variables are statistically independent, and conversely, the higher the AMI between them, the more information each contains about the other. In the context of this work, AMI provides a useful index relating movements of the head to those of the articulators, with higher values associated with greater mutual dependency. We computed AMI on the first principal component by epoch for the pairs HEAD:ART1 (MIH1), HEAD:ART2A (MIH2A), HEAD:ART2B (MIH2B), and HEAD:JAW (MIHJ), where ART1 was the non-alternating (syllable-rate) articulator trajectory (e.g., LA in *to**p** c**op***), ART2A was the first alternating (half syllable-rate) articulator of the pair (e.g., TT in ***t**op cop*), and ART2B was the second alternating articulator of the pair (e.g., TD in *top **c**op*). For non-alternating control pairs, ART1 was the coda trajectory, and both ART2A and ART2B were mapped to the onset trajectory. Table 3 provides a glossary of these relationships.

##### Mutual Power

**FIGURE 3:**
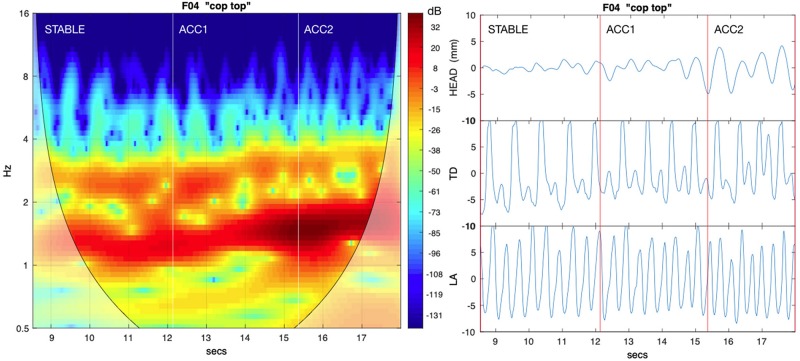
The **left panel** shows mutual power (MP) between HEAD and tongue dorsum (TD) for an exemplar *cop top* sequence. Increasingly darker red hues indicate higher values of MP; lighter shades to the lower left and right indicate possible wavelet edge effects. The **right panel** shows corresponding source PC1 trajectories for HEAD, alternating /k/ (TD) and syllable rate /p/ lip aperture (LA). Both the syllable rate and alternating rate frequency bands show significant MP, but highest values are observed at the lower alternating frequency, showing that is the base rate for HEAD movement.

Entrainment between the head and the speech articulators can also be investigated using estimates of *mutual power* (MP) in the alternating and non-alternating frequency bands. It was computed here using the cross-wavelet transform (Grinsted et al., 2004), which convolves the discrete wavelet transform of one signal with the complex conjugate of the other, with MP given by the absolute value of the result converted to dB. This is a spectral representation similar to a spectrogram in which successive frames (time) encode power at different frequencies, with MP highest for those frequencies which are mutually coherent between the paired trajectories. Figure 3 provides an example pairing HEAD and TD for a *cop top* sequence, showing relative MP in the alternating and non-alternating frequency bands. We used the Cross Wavelet Toolbox (Grinsted, 2014) to compute MP by epoch for the same first principal component pairs used to measure AMI. To quantify MP over each epoch, we tracked resonance amplitude peaks for the frequency band closest to both the expected syllable and alternating rates (as determined by the mean metronome click rate for the epoch) and determined their median values. For the HEAD:ART1 comparison, MPH1**1** represents the median value in the syllable (non-alternating) frequency band, and MPH1**2** represents the median value in the alternating band. Similarly, for the HEAD:JAW comparison, MPHJ1 and MPHJ2 give power in the syllable and alternating frequency bands, respectively. For the HEAD:ART2A comparison, MPH2A1 and MPH2A2 give the syllable and alternating frequency band values, and likewise for the HEAD:ART2B comparison, MPH2B1 and MPH2B2 give the syllable and alternating frequency band values. As with AMI, for non-alternating control pairs ART1 was the coda trajectory, and both ART2A and ART2B were mapped to the onset trajectory. See Table 3 for a glossary of these relationships.

#### Error-Based Measures

To investigate whether head movement is locally sensitive to error occurrence, we examined its peak velocity (EPV) immediately preceding and following each error. The PRE and POST evaluation windows for comparison were set equal to twice the length of the metronome click period containing the error; that is, for an error occurring at time *t* within a click period of duration *p*, the PRE value for that error was the peak HEAD speed achieved over the *t–p* range paired with the POST value over the *t*+*p* range. HEAD speed was computed as the tangential velocity of the UI sensor trajectory using central differencing.

### Analysis

Statistical analysis of the collected data was performed within the *R* environment (R Core Team, 2018). Effect sizes for paired *t*-tests were evaluated using Cohen’s *d* statistic. Linear mixed-effects models were evaluated using the *lme4* (Bates et al., 2015) and *lmerTest* (Kuznetsova et al., 2017) packages. Log-likelihood comparisons were used to assess whether interaction terms and random slopes by speaker and word pair were supported. Significance of model fixed effects was assessed using estimates of the regression coefficients divided by their SEs (a *t*-test), with degrees of freedom based on the Satterthwaite approximation. Model effect sizes were evaluated using partial *R*^2^, the proportion of variance explained by the fixed effects alone, and conditional *R*^2^, the proportion of variance explained by both fixed and random effects, using the methods of Nakagawa and Schielzeth (2013). Significant results are indicated using the *p* < 0.001 ^∗∗∗^, *p* < 0.01 ^∗∗^, *p* < 0.05 ^∗^, and *p* < 0.10 • convention. Full model outputs (indexed as M1, M2, …below) are provided as Supplementary Material. Note that we do not consider possible lexical effects because the task used common real words of English with simple CVC structure and because we consider that the nature of the task (rote repetition) minimizes lexical influence following the first production instance.

## Results

### Error Rates

**FIGURE 4:**
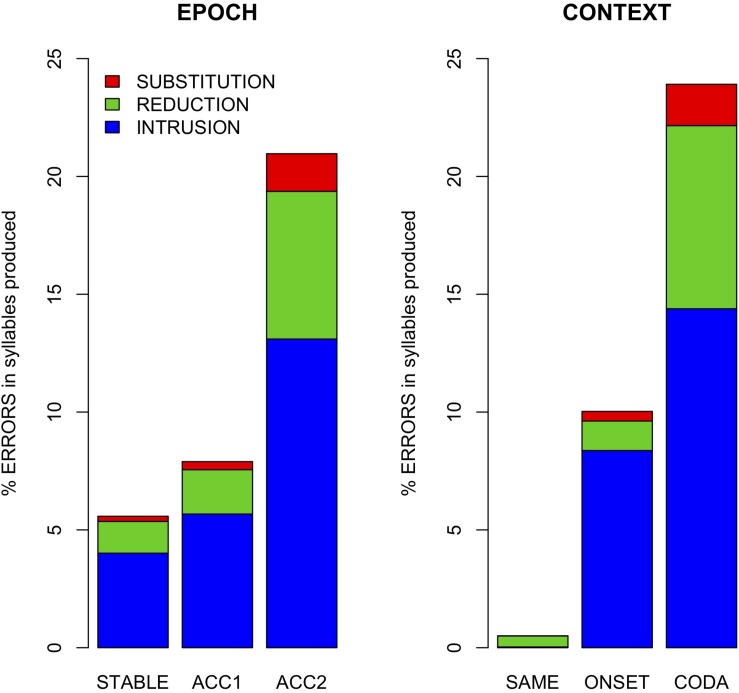
Error rates by epoch and context across all speakers.

Table 2 summarizes error counts by speaker and conditions, and Figure 4 shows their distribution as error rates normalized by the number of syllables produced per epoch. As shown in Figure 4, error rate was affected by both context (alternation task) and production rate (epoch). Gestural intrusion (co-constriction of the anti-phase articulator) was the most common type of error. Extending the results of Slis and Van Lieshout (2016) to coda alternation, most intrusive errors were produced with the TD articulator and the fewest with the lips. A model (M1) predicting error rate (combined across all types) by fixed effects of context and epoch and their interaction, with random intercepts by speaker and word pair, showed a significantly greater main effect for context ONSET (*t* = 2.1 ^∗^) and CODA (*t* = 3.0 ^∗∗^) alternation than for no alternation (SAME). While no main effect of epoch was observed, its interaction with context showed significantly higher error rates in the accelerated epoch ACC2 for alternating trials (ACC2:ONSET *t* = 3.8 ^∗∗∗^, ACC2:CODA *t* = 9.8 ^∗∗∗^). For this model, partial *R^2^* = 0.37, conditional *R^2^* = 0.49.

**TABLE 2:** Error counts by speaker and condition [error types: intrusions, reductions, substitutions; context: same, onset, coda alternation; epoch: stable, initial, and final accelerating production rates; and articulator: tongue dorsum (TD), tongue tip (TT), lip aperture (LA)].

**TYPE**	**F23**	**F24**	**F29**	**F33**	**F34**	**M25**	**M28**	**M32**	**M35**	**Totals**
(ALL)	95	42	157	109	223	139	183	119	32	1,099
INT	48	30	89	79	159	102	113	81	26	727
RED	35	12	47	25	53	31	60	35	5	303
SUB	12	0	21	5	11	6	10	3	1	69

	**SAME**	**ONSET**	**CODA**	**STABLE**	**ACC1**	**ACC2**	**TD**	**TT**	**LA**	

(ALL)	16	320	763	178	252	669	406	418	206	
INT	1	267	459	128	181	418	298	270	159	
RED	15	40	248	43	60	200	108	148	47	
SUB	0	13	56	7	11	51	–	–	–	

**TABLE 3:** Glossary of dependent variables.

**Variable**	**Pairing**	**Description**
MVT	–	Distance (path integral) traveled by HEAD during epoch, scaled by epoch duration
VEL	–	Max over epoch of peak HEAD speed over metronome periods scaled by period durations
EPV	–	Peak HEAD speed over local interval (twice metronome period) preceding/following error
MIHJ	HEAD: JAW	AMI between PC1 of HEAD and JAW
MIH1	HEAD: ART1	AMI between PC1 of HEAD and non-alternating articulator (ART1; e.g., LA in *top cop*)
MIH2A	HEAD: ART2A	AMI between PC1 of HEAD and 1st alternating articulator (ART2A; e.g., TT in *top cop*)
MIH2B	HEAD: ART2B	AMI between PC1 of HEAD and 2nd alternating articulator (ART2B; e.g., TD in *top cop*)
MPHJ1	HEAD: JAW	MP between PC1 of HEAD and JAW at syllable rate frequency
MPHJ2	HEAD: JAW	MP between PC1 of HEAD and JAW at alternating rate frequency
MPH11	HEAD: ART1	MP between PC1 of HEAD and non-alternating articulator at syllable rate frequency
MPH12	HEAD: ART1	MP between PC1 of HEAD and non-alternating articulator at alternating rate frequency
MPH2A1	HEAD: ART2A	MP between PC1 of HEAD and 1st alternating articulator at syllable rate frequency
MPH2A2	HEAD: ART2A	MP between PC1 of HEAD and 1st alternating articulator at alternating rate frequency
MPH2B1	HEAD: ART2B	MP between PC1 of HEAD and 2nd alternating articulator at syllable rate frequency
MPH2B2	HEAD: ART2B	MP between PC1 of HEAD and 2nd alternating articulator at alternating rate frequency

*PC1, first principal component; AMI, average mutual information; MP, mutual power. For non-alternating control pairs, ART1 is mapped to the coda articulator, and ART2A and ART2B to the onset articulator.*

### Head Movement

**FIGURE 5:**
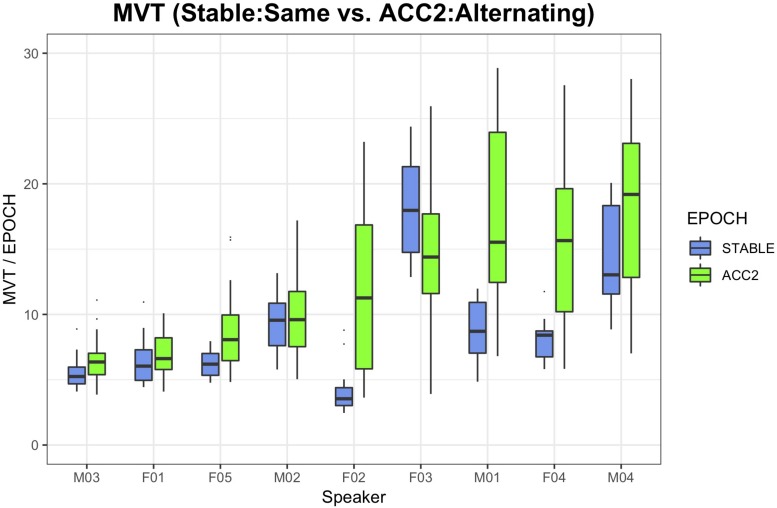
Boxplots of head movement by speaker, contrasting the condition with least expected movement (initial stable epoch, same word context) with the most (ACC2 epoch, alternating words), sorted by magnitude of ACC2 movement.

Figure 5 illustrates the range of observed head movement by speaker, contrasting the STABLE:SAME condition, where least movement is expected, to the ACC2:ONSET,CODA (alternating) conditions where the most movement is expected. With two exceptions (M02 and F03, who showed head movement across all conditions), the accelerating metronome task resulted in increased mean head movement by epoch.

To adjust for a left-skewed distribution, head MVT measures were log-transformed for analysis. In addition to fixed effects of context and epoch, a derived *error* factor was used to distinguish between error-free epochs (ERROR = F) and epochs in which at least one labeled speech error occurred (ERROR = T). A model (M2) predicting log(MVT) from fixed effects of epoch and error, including random slopes for error by speaker and random intercepts by word pair, showed marginally more movement for errorful epochs overall (*t* = 2.0 •) and significantly more movement for the accelerating epochs ACC1 (*t* = 2.2 ^∗^) and ACC2 (*t* = 5.8 ^∗∗∗^) than the baseline stable epoch (inclusion of their interaction and an effect of alternation context were unsupported by model comparison). Partial *R^2^* = 0.04, conditional *R^2^* = 0.52.

### Head Peak Velocity

#### Evaluated by Epoch

**FIGURE 6:**
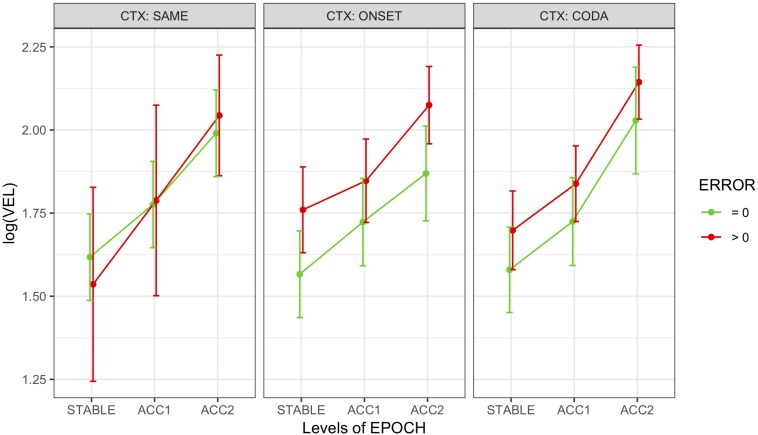
Marginal means by condition for epoch-based head peak velocity (VEL) grouped over speakers and distinguishing error-free trials from those with at least one error. Error bars show SEM.

Head peak velocity measures (VEL) were also left-skewed and thus log-transformed for analysis. Figure 6 shows log(VEL) means and their SEs by epoch, context, and error grouped across speakers. Model comparison for the epoch-based measures resulted in a comparable model (M3) to that used for movement analysis, predicting log(VEL) from fixed effects of epoch and error with random slopes for error by speaker and random intercepts by word pair, with no interaction and no effect for context. The pattern of results was similar to that found for movement, showing marginally higher peak velocity for errorful epochs overall (*t* = 2.2 •) and significantly higher within the accelerating epochs ACC1 (*t* = 4.8 ^∗∗∗^) and ACC2 (*t* = 12.3 ^∗∗∗^). Partial *R^2^* = 0.12, conditional *R^2^* = 0.54. A *post hoc* test (Tukey HSD) confirmed that log(VEL) was significantly different by epoch, ordered as STABLE < ACC1 < ACC2 at the *p* < 0.0001 level (adjusted).

#### Evaluated Over Local Error Neighborhood

Head peak velocity evaluated over the local PRE/POST neighborhood for each error provides twinned measurements suitable for a one-sided (*H*_1_: POST > PRE) paired *t*-test. The results show clearly that, in general, head peak velocity increases immediately following errors: *t* (1,098) = 6.5 ^∗∗∗^; Cohen’s *d* = 0.2. A model (M4) predicting error-local log(VEL) with fixed effects of epoch, context, and PP (PRE/POST), with random intercepts by speaker and word pair, confirmed that POST > PRE (*t* = 3.3 ^∗∗∗^). Interactions were not supported. Main effects for epoch showed greater peak velocity associated with errors in the ACC2 condition (*t* = 2.9 ^∗∗^) and with ONSET (*t* = 2.4 ^∗^) and CODA (*t* = 2.9 ^∗∗∗^) alternation. Partial *R^2^* = 0.01, conditional *R^2^* = 0.41.

To investigate the possibility that the onset of head movement triggered by errors might be sensitive to the either the *type* of error (i.e., reduction or intrusion) or the active articulator (TD, TT, and LA), an additional model (M5) was fit, predicting error-local log(VEL) from fixed effects of context, error type, articulator, and PP, with random slopes for context and type by speaker and random intercepts by word pair. To reduce the complexity of the analysis, the subset of data used with this model excluded substitutions and the non-alternating (SAME) context given the low and unbalanced error rate in that condition (15 reductions but just one intrusion and no substitutions; Table 2) and did not include EPOCH as a fixed effect on the reasoning that the comparison PRE/POST error was valid regardless of the epoch within which it occurred. Interactions between context and error type and between context and articulator were supported, but not with PP. Model results show that head peak velocity increases: immediately following errors (POST > PRE; *t* = 3.6 ^∗∗∗^); more for reductions (*t* = 3.0 ^∗^), although this is offset in coda alternation (*t* = −2.7 ^∗^); and more for TD (*t* = 2.7 ^∗∗^) and TT (*t* = 2.5 ^∗^) articulators, again offset in coda alternation (*t* = −2.7 ^∗∗^, *t* = −3.1 ^∗∗^). *Post hoc* tests (Tukey HSD) confirmed RED > INT and TD, TT > LA at the *p* < 0.05 level for onset contexts; not significant (n.s.) for coda contexts. Partial *R^2^* = 0.02, conditional *R^2^* = 0.49.

### Average Mutual Information

Recall that AMI was computed pairwise between HEAD and the non-alternating (syllable rate) articulator ART1 (MIH1), the first alternating articulator ART2A (MIH2A), the second alternating articulator ART2B (MIH2B), and JAW (MIHJ). For non-alternating control pairs, ART1 was the coda trajectory, and both ART2A and ART2B were mapped to the onset trajectory. To assess whether more information is shared between HEAD and the alternating articulators rather than the non-alternating articulator, as a first analysis, one-sided (*H*_1_: MIH2A, B > MIH1) paired *t*-tests were performed on the alternating (context = ONSET, CODA) trials alone. For both ART2A (MIH2A > MIH1: *t* (665) = 14.5 ^∗∗∗^, Cohen’s *d* = 0.6) and ART2B (MIH2B > MIH1: *t* (665) = 15.6 ^∗∗∗^, Cohen’s *d* = 0.6), results confirm greater entrainment of HEAD with the alternating articulators, while a two-sided paired *t*-test found no significant difference between the first and second alternating articulators (MIH2A ≠ MIH2B: *t* (665) = 1.3 n.s.).

**FIGURE 7:**
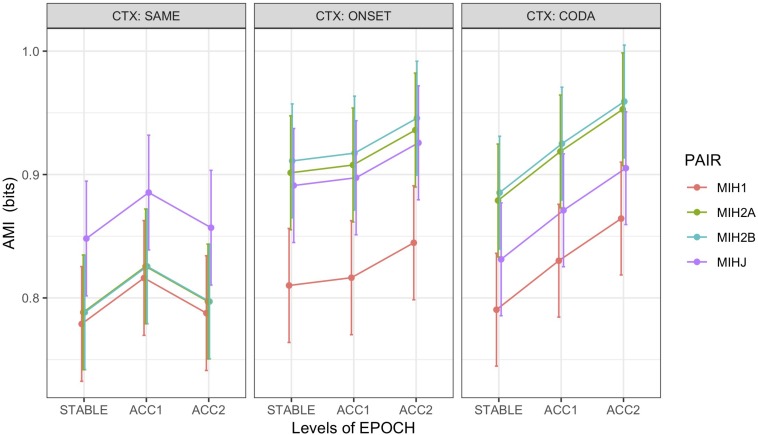
Marginal means by condition for average mutual information (AMI) between PC1 of HEAD with non-alternating articulator (MIH1), first (MIH2A) and second (MIH2B) alternating articulators, and JAW (MIHJ), grouped over speakers and including errors. For non-alternating control pairs (CTX:SAME), ART1 was the coda trajectory, and both ART2A and ART2B were mapped to the onset trajectory. Error bars show SEM.

An additional analysis on all word pairs including the non-alternating controls was performed using a linear mixed-effects model (M6) predicting AMI from fixed effects of epoch, context, and a derived variable *pair* encoding the HEAD-paired articulator, with random intercepts by speaker and word pair. Model comparison supported inclusion of interaction terms for epoch:context and context:pair, but not an effect for error. Figure 7 illustrates marginal means for this model. Results showed main effects of significantly greater AMI between HEAD and JAW than the HEAD:ART1 baseline (*t* = 5.8 ^∗∗∗^) and for the first acceleration (ACC1) epoch than the initial stable epoch (*t* = 3.6 ^∗∗∗^). AMI significantly increased in the second acceleration (ACC2) epoch only under alternation, with CODA increasing more than ONSET (ACC2:ONSET *t* = 1.8 •, ACC2:CODA *t* = 4.9 ^∗∗∗^). As is evident from Figure 7, the interaction between context and pair was driven by significantly higher AMI between HEAD and both alternating articulators in the alternating *vs.* non-alternating (SAME) contexts (ONSET:MIH2A *t* = 5.0 ^∗∗∗^, CODA:MIH2A *t* = 5.1 ^∗∗∗^, ONSET:MIH2B *t* = 5.6 ^∗∗∗^, CODA:MIH2B *t* = 5.6 ^∗∗∗^). For this model, partial *R^2^* = 0.07, conditional *R^2^* = 0.55. *Post hoc* tests (Tukey HSD) found no difference in AMI between HEAD paired with either the onset (MIH2A, MIH2B) or coda (MIH1) of non-alternating control pairs but confirmed the hierarchy MIH2A, MIH2B, MIHJ > MIH1 for both ONSET and CODA alternating contexts (*p* < 0.0001). In addition, in CODA contexts, MIHJ was significantly ordered *between* MIH2A,B and MIH1 (i.e., MIH2A, MIH2B > MIHJ > MIH1; *p* < 0.0001), indicating that biomechanical coupling between the head and jaw is insufficient to account for the degree of observed entrainment between HEAD and the alternating articulators.

### Mutual Power

As with AMI, MP was computed pairwise between HEAD and the non-alternating articulator ART1 (MPH1*x*), the first and second alternating articulators ART2A and ART2B (MPH2A*x*, MPH2B*x*) and the jaw (MPHJ*x*). For non-alternating control pairs, ART1 was the coda trajectory, and both ART2A and ART2B were mapped to the onset trajectory. MP was assessed for each pairing in the syllable rate frequency band (*x* = 1) and the alternating rate frequency band (*x* = 2); for example, MPH2A**2** gives MP between HEAD and ART2A in the alternating frequency band.

To test whether the head moved at a frequency tracking the alternating rather than the non-alternating articulator, reflected in higher MP observed at the slower rate, a one-sided (*H*_1_: MPH2A2, MPH2B2 > MPH11) paired *t*-test was applied to the alternating (context = ONSET, CODA) trials alone. The results strongly support the hypothesis, showing that substantially higher power was observed in the alternating frequency band for both the HEAD:ART2A and ART2B pairings than the syllable rate HEAD:ART1 comparison (MPH2A > MPH11: *t* (665) = 19.2 ^∗∗∗^, Cohen’s *d* = 0.7, MPH2B > MPH11: *t* (665) = 19.4 ^∗∗∗^, Cohen’s *d* = 0.8). An additional two-sided paired *t*-test found no significant difference between the first and second alternating articulators (MPH2A2 ≠ MPH2B2: *t* (665) = 0.9 n.s.).

A confirmatory analysis (M7) was performed on the alternating word pairs to predict MP from fixed effects of epoch, context, error, and PAIR, with pairings MPH11, MPH2A2, and MPH2B2. Model comparison supported the inclusion of an interaction term between error and context, random intercepts by speaker, and random slopes for pair by word. Results showed an increase in MP for errorful trials (*t* = 3.0 ^∗∗^), although this was decreased in coda contexts (*t* = −2.6 ^∗^). The pairings of HEAD with the alternating rate articulators (MPH2A2: *t* = 8.5 ^∗∗∗^, MPH2B2: *t* = 8.5 ^∗∗∗^) showed overwhelmingly greater MP (at the alternating rate) than the baseline syllable rate articulator MPH11, confirmed by *post hoc* (Tukey HSD) tests at the adjusted *p* < 0.0001 level, which also found no significant difference between MPH2A2 and MPH2B2. Partial *R^2^* = 0.18, conditional *R^2^* = 0.47. The model also showed that MP was significantly reduced in the fastest epoch ACC2 (*t* = −6.7 ^∗∗∗^). This result may be due to a loss of systematic coherence or increased production variability as errors multiply under rate pressure, since MP amplitude is affected by any deviation from expected alternation frequencies. As observed error rate is highest in ACC2 epochs and CODA alternation contexts, the lower MP values for those conditions may reflect error-driven deviation from the alternating rate, particularly if a frequency reorganization like that shown in Figure 9 occurs. Conversely, the higher value seen overall for MP in errorful epochs likely reflects the increase in head movement observed in the MVT and VEL results; if such movement continues to track the alternation frequency, as in the Figure 10 example, then higher coherent MP is to be expected.

Both AMI and MP results to this point show the head coupled with movement of the alternating articulators and with highest MP at the alternating frequency (although MP evaluated on alternating contexts only). However, this coupling may arise from two as yet undifferentiated sources. One possibility is that speakers may use the head to signal prosodic stress on each pair, for example, ***tóp** cop* or *top **cóp***. In this case (*H*_*A*_), MP between HEAD and either articulator in the non-alternating control pairs should be highest at the frequency of prosodic alternation driving the head; that is, strongest at the alternation frequency regardless of context. An alternative possibility is that this coupling reflects reinforcement of the executing motor plan for the less stable (1:2) alternating word pairs only, as necessitated by increasing rate pressure. In this case (*H*_*B*_) MP for the non-alternating controls should be highest at the syllable rate because recruitment of the head is either unnecessary given the more stable (1:1) production pattern or if recruited tracks the 1:1 frequency.

**FIGURE 8:**
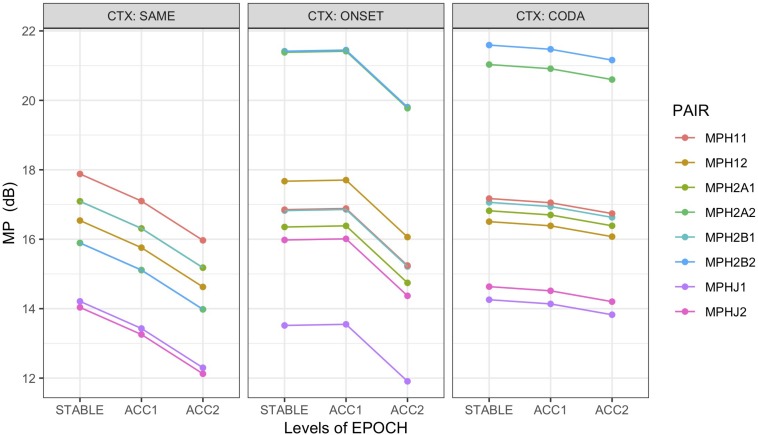
Marginal means by condition for mutual power (MP) between PC1 of HEAD with non-alternating articulator (MIH1x), first (MIH2Ax) and second (MIH2Bx) alternating articulators, and JAW (MIHJx), grouped over speakers. MP assessed at syllable rate (*x* = 1) and alternating rate (*x* = 2). Trials with errors excluded from analysis. For non-alternating control pairs (CTX:SAME), ART1 was the coda trajectory, and both ART2A and ART2B were mapped to the onset trajectory. Error bars suppressed for legibility.

To distinguish between these possibilities, a linear mixed-effects model (M8) that included the non-alternating controls was used to predict MP from fixed effects of epoch, context, and PAIR, with random intercepts by speaker and word pair. Pairings of HEAD with ART1, ART2A, ART2B, and JAW were included at both the syllabic and alternating frequency rates. Recall that for non-alternating control pairs ART1 was the coda trajectory and both ART2A and ART2B were mapped to the onset trajectory. Only error-free epochs were used (Ns: SAME = 229, ONSET = 155, CODA = 138) to avoid the phase-resetting disruptions of errors on the computation of MP. Model comparison supported the inclusion of interaction terms for epoch:context and context:pair. Figure 8 illustrates the marginal means for this model. It is readily apparent from this figure that in the control (context:SAME) condition, the strongest mutual power is between the head and the ART1 (coda) trajectories at the syllabic rate (MPH11), well above MP at the alternating rate (MPH12), whereas in the alternating (ONSET, CODA) contexts, highest MP occurs at the alternating frequency rate (MPH2A2, MPH2B2), thus confirming *H*_*B*_. As quantified by the model, all pairings for the STABLE context have lower MP than the MPH11 baseline: MPH12 *t* = −3.3 ^∗∗∗^, MPH2A1 *t* = −1.9 •, MPH2A2 *t* = −4.9 ^∗∗∗^ (Recall that MPH2B is a copy of MPH2A in STABLE contexts). Tukey HSD contrasts averaged over EPOCH for the STABLE context have the ordering MPH11, MPH2A1 > MPH12, MPH2A2 > MPHJ1, MPHJ2, significant at an adjusted value of *p* < 0.02. In the interaction of pairing with context, however, both first and second alternating articulators show strongest MP at the alternating rate, overwhelmingly greater than the MPH11 baseline (ONSET:MPH2A2 *t* = 10.2 ^∗∗∗^, CODA:MPH2A2 *t* = 8.9 ^∗∗∗^, ONSET:MPH2B2 *t* = 10.3 ^∗∗∗^, CODA:MPH2B2 *t* = 9.7 ^∗∗∗^). Partial *R^2^* = 0.18, conditional *R^2^* = 0.42. The pairing of HEAD with JAW shows the least energy for all three contexts, in both frequency bands, demonstrating again that it is not the underlying driver of head movement. As in the simpler model, an effect of epoch shows that MP declines as rate increases (subject to interaction with context), with the lowest values found at the fastest rate (main effect ACC2 *t* = −7.7 ^∗∗∗^). Because errors were not included in this analysis, this result is likely due to loss of coherence (thus affecting MP) as accelerating production rate leads to greater variability in the articulation of each sequence.

### Summary of Results

*Error Rates* (Figure 4): More errors were observed in alternation conditions (CODA > ONSET > SAME) and at faster production rates (ACC2 > ACC1 > STABLE). Intrusions were most common (66%), followed by reductions (28%) and substitutions (6%). For intrusions, TD was the most common articulator (41%), followed by TT (37%) and LA (22%). For reductions, TT was most common (49%), followed by TD (36%), and LA (15%).

*Head Movement* (Figure 5): Increased by epoch with production rate.

*Head Peak Velocity* (Figure 6): By epoch, increased with production rate. By local error, uniformly increased immediately following the error (POST > PRE); in ONSET alternation contexts, reductions increased more than intrusions and TD and TT articulators more than LA (in CODA alternation, these contrasts were n.s.).

*Average Mutual Information* (Figure 7): Greatest MI observed between head and the alternating (phrasal rate) articulators (MIH2A|B), least between head and the non-alternating (syllable rate) articulator (MIH1), and intermediate MI between head and jaw (MIHJ).

*Mutual Power* (Figure 8): For alternating trials only, including errors, highest MP was observed at the alternating (phrasal) rate; this increases in errorful trials and is reduced in CODA alternation and ACC2 epochs. For all trials, including non-alternating controls and excluding errors (to test possible effects of prosodic stress), no significant MP was found at the alternating frequency for controls but significant power at that frequency for alternating trials.

## Discussion

The pattern of observed speech errors increasing by epoch demonstrates the effectiveness of the accelerating rate task for imposing pressure on production and confirms that errors occur more frequently in coda than in onset alternation. Returning to the questions raised in the *Introduction*, the results show clearly that head movement, as indexed by distance traveled (MVT) and peak velocity aggregated over epochs (VEL), does increase with speech production rate as driven by the increasing rate metronome. Head movement was also significantly greater within epochs in which at least one error occurred compared to error-free production. In addition, peak velocity was shown to increase significantly immediately following labeled production errors, thus confirming the previous observations of Dittmann and Llewellyn (1969) and Hadar et al. (1984b). Some effects of error type were seen: more intrusions than reductions or substitutions were obtained overall, and more intrusions occurred with the TD articulator than with TT or LA, confirming the pattern reported by Slis and Van Lieshout (2016). While both AMI and MP results show significant coupling of the head to the jaw, this was in both cases subordinate to that seen for the pairing of the head to the constriction-forming articulators, thus ruling out the jaw as a primary source driving the entrainment (The lesser magnitude coupling that does exist between head and jaw likely arises from its synergistic role in helping form the constrictions).

The question of whether head movement is sensitive to onset *vs.* coda asymmetry has a more nuanced answer. Neither MVT nor VEL supported an effect of alternation context in modeling. However, AMI computed between HEAD and the alternating and syllable-rate articulators showed an interaction between epoch and context such that the overall effect of increased AMI in the fast rate epoch ACC2 was significantly enhanced in the CODA alternation condition. As AMI requires some minimal level of systematic head movement to predict the paired articulator movement effectively, it is unsurprising that it should be greater in the ACC2 epoch with largest observed head movement. Also, given the higher overall error rates seen in the CODA alternation context, and based on the longer production times reported for CODA alternation by Sevald and Dell (1994), it is likely that the ACC2:CODA condition was the most difficult for speakers to execute. If the head is recruited to facilitate production under increasing pressure, then this condition is also the most likely to show the greatest entrained coordination between paired articulators as reflected by AMI, explaining the observed interaction. The reason that no corresponding effect of context was observed for head movement alone may derive from a lack of sufficient sensitivity: as shown by the spontaneous increase in movement observed following errors, any epoch that includes them will show greater movement overall, swamping any effect of context.

The AMI and MP results for the alternating context conditions clearly show that when the head does move, it tracks the alternating (phrasal) rate rather than the syllable-rate frequency, reflected in the highest values seen for these measures in the pairings between HEAD and both alternating articulators. Because these measures are computed in very different ways, their converging confirmation of this behavior is especially significant. We have considered two possibilities for why the head preferentially moves at the alternating frequency rate. Under the first, head movement is reflecting an imposed phrasal stress pattern, as in trochaic “***tóp** cop, **tóp** cop*.” Were such to be the case however, it should also apply consistently to control sequences like “***tóp** top, **tóp** top*” and result in high MP at the alternating rate for those trials as well. However, results for control trial sequences instead show highest MP at the syllable rate, undermining this explanation. The alternative, supporting hypothesis *H*_1_, is that the head is recruited for enhancing stability of the 1:2 alternation pattern as production difficulty increases, a point considered more fully below.

It is possible that additional factors, not considered in this study, may also play a role in driving head movement. For example, conscious awareness of errorful production has been shown to lead to more dynamic facial expression (Barkhuysen et al., 2005), and this may in turn be coupled with increased head movement. Speakers may also have been distracted or influenced by the presence of the experimenters observing their production and used movement of the head in a communicative mode to signal correction following self-perceived errors. Future studies should consider recording facial features and polling participants for their self-awareness of errors to address these concerns. However, because head movement was observed to increase systematically with rate pressure even without errors, self-awareness alone seems unlikely to be its primary cause.

In summary then, speaker head nodding increased with production effort and increased abruptly following errors. More errors were observed under faster production rates and in coda rather than onset alternations. More intrusions were observed than reductions or substitutions, and more errors were produced with the tongue (TD, TT) than the lips (LA). Neither jaw coupling nor imposed prosodic stress was observed to be a primary driver of head movement. The greatest entrainment between head and articulators was observed at the fastest rate under coda alternation. And nodding frequency in alternating word pairs tracked the alternation rate rather than the syllable rate. But these results leave open the additional question of *why* the head or other extremities should be systematically related to articulatory movement.

The study by Hadar (1991) mentioned above measured the head movement of aphasics and normal controls engaged in speech during interviews, finding that while head movement was positively correlated with speaking rate for both groups, it was highest for non-fluent aphasic speakers, who apart from speech coordination difficulties showed no other motor impairment. In a different domain, Goebl and Palmer (2009) showed that pianists performing a duet with manipulated auditory feedback increased the magnitude and coherence of their head movements as this feedback was degraded. In both cases, head movement appears to be supplemental to normal patterns of movement compensating for some kind of stress or impairment. Moreover, studies of dual-task demands imposed by walking and talking simultaneously (e.g., Kemper et al., 2003) show that when the head is unavailable for recruitment (because of its role in maintaining balance), both speech rate and fluency decline, particularly in older adults.

**FIGURE 9:**
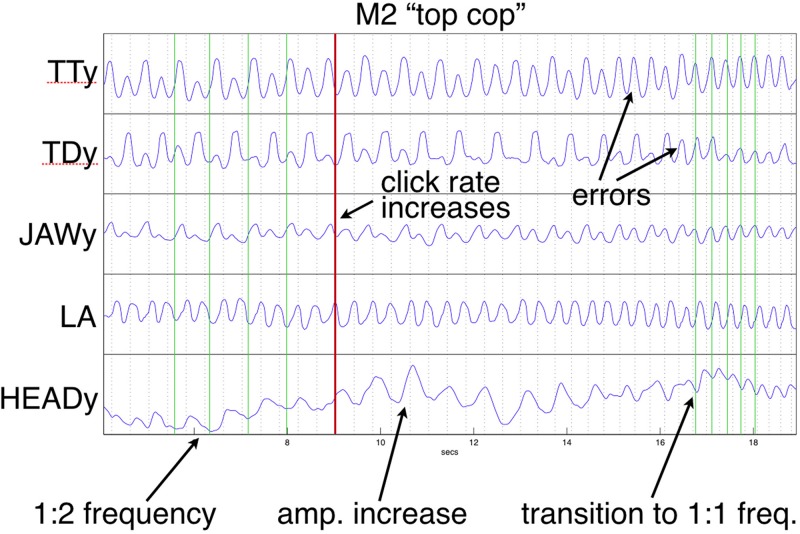
Frequency reorganization example: Head movement increases as metronome rate accelerates; at the highest rate, all articulators, including the head, have entrained 1:1 to the syllabic rate. Except for lip aperture (LA), trajectories show vertical components of movement. Green vertical lines index HEAD minima [compare with alternating tongue tip (TT) and tongue dorsum (TD)].

**FIGURE 10:**
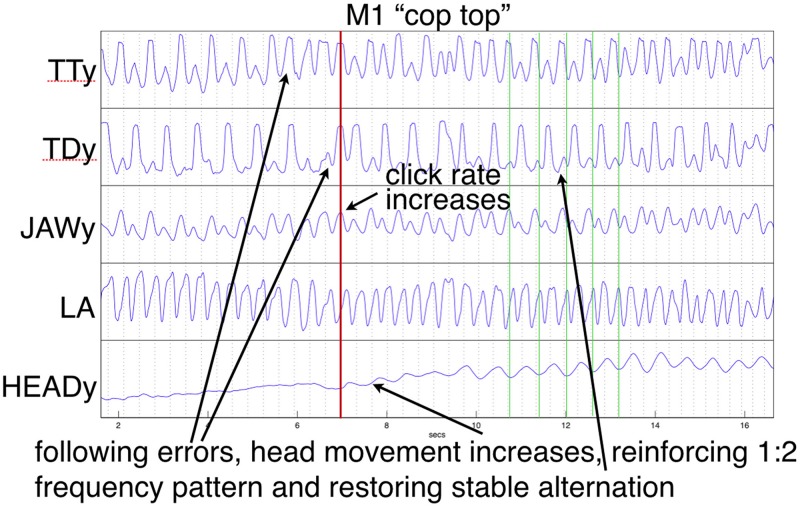
Example of production errors followed by increased head movement that eventually tracks and stabilizes the alternation frequency. Except for LA, trajectories show vertical components of movement. Green vertical lines index HEAD minima (compare with alternating TT and TD).

In the current study, the “*cop top*” trial shown in Figure 10 provides a relevant example. Initially, the head is almost still, but it begins to move following a series of errors, eventually tracking the TT constriction as error-free alternation is (temporarily) restored. This illustrates a previously mentioned explanation, the recruitment of additional degrees of freedom to reinforce a (wobbly) coordinative structure in its execution of a motor pattern. As discussed above, the particular pattern arising from word pair alternation requires reinforcement because of its juxtaposition of syllabically *vs.* bisyllabically recurring articulation in a 1:2 frequency ratio, which is less stable than a 1:1 relationship, especially under rate pressure (Haken et al., 1985; Kelso et al., 1993; Goldstein et al., 2007). The “*top cop*” trial shown in Figure 9 provides an example of what happens when production rate becomes overwhelming: an increase in head nodding magnitude at the alternating frequency following initial rate acceleration is ultimately insufficient to prevent a phase transition that leaves all articulators including the head oscillating at the 1:1 syllabic frequency.

While the trials shown in Figures 9, 10 represent interesting examples, in most cases though, recruitment of the head (and, although not recorded, the feet and hands, which were also sometimes observed to tap at the alternation frequency) served to stabilize the coordinative structure assembled to articulate the speech task under increasing rate pressure. Because the alternation frequency is less stable than the base syllable rate when words within the pair differ, crucially, that is the rate that the head was observed to support. When as in the example shown in Figure 10 this recruitment follows immediately upon a production error, the reorganization of the coordinative structure to include the head appears to act to reset and restore the appropriate phase relations among articulators. As expressed by Kelso et al. (1993, p. 365):

“[A] system containing a set of active components that have been self-organized for a particular movement pattern is […] no longer able to support that behavior in a stable fashion when a control parameter (here the frequency of motion) crosses a critical value. The new movement pattern may still be topologically equivalent to the previous one […] but additional d.f. are required to perform the task.”

In general, the recruitment of additional degrees of freedom is directly related to maintaining the executing task, as for example when both hands are needed to stabilize manipulation of a significant weight. What is interesting about head nodding, foot tapping, and other peripheral extremities recruited as in this task to maintain a rhythmic pattern under production stress is that they are at best only very loosely related biomechanically to the actual articulation of speech. The Coupling Graph model (Nam et al., 2009) predicts that the more connections that exist between the oscillators that collectively produce speech gestures, the more stable the relationships between those oscillators will be. Entrained oscillation of the head, despite contributing little or nothing directly to articulation, nonetheless serves in this view as a contributor to overall stability of the executing motor plan. Our results, particularly the abrupt increase in head movement observed following errors, provide evidence in support of coupling graph reorganization to include the head for this purpose. Thus, while under normal speaking conditions, the primary function of head movement is communicative, this work shows that head movement in speech tasks can also be driven by motoric influences, and that its recruitment can serve as a means of preserving articulatory stability under production duress.

## Data Availability Statement

The raw data supporting the conclusions of this manuscript will be made available by the authors, without undue reservation, to any qualified researcher upon request.

## Ethics Statement

This study was carried out in accordance with the recommendations of Yale Institutional Review Board with written informed consent from all subjects, who were paid for their participation. All subjects gave written informed consent in accordance with the Declaration of Helsinki. The protocol was approved by the Yale Institutional Review Board.

## Author Contributions

MT, CM, and LG designed the experiment and wrote the manuscript. MT supervised the data collection and performed the data analysis. CM supervised the error labeling.

## Conflict of Interest

The authors declare that the research was conducted in the absence of any commercial or financial relationships that could be construed as a potential conflict of interest.
