# Supplementary material for: Noggin Nodding: Head Movement Correlates With Increased Effort in Accelerating Speech Production Tasks
Source: Front Psychol. 2019 Nov 27;10:2459. doi: 10.3389/fpsyg.2019.02459 (PMC6890824; doi:10.3389/fpsyg.2019.02459)
Supplement: Supplementary file 1 [file Data_Sheet_1.PDF]

Supplemental Material

Linear Mixed-Effects Models supporting “Noggin nodding: Head movement correlates with increased effort in accelerating speech production tasks”

Data summary

By epoch

```
       ID             WORD          CTX           EPOCH         ERROR
F04     :129   TAKTAP : 39   SAME :240    STABLE:302    FALSE:522
F05     :129   TAPTAK : 39   ONSET:285    ACC1  :302    TRUE :384
F03     :114   TAKTAK : 30   CODA :381    ACC2  :302
M02     :114   TAPTAP : 30
M03     :114   KODKOB : 27
F01     : 90   KODPOD : 27
(Other):216   (Other):714
```

By local error

```
       ID          CTX           EPOCH         TYPE       ART
F05     :446   SAME :  32   STABLE: 356   INT:1454   LA:412
M02     :366   ONSET: 640   ACC1  : 504   RED: 606   TD:812
F03     :314   CODA :1526   ACC2  :1338   SUB: 138   TT:836
M01     :278                                         XX:138
M03     :238
F04     :218
(Other):338
```

Model M1: error rate

Predict error rate (ER) from fixed effects of context and epoch and their interaction, with random intercepts by speaker ID and word pair

```
Linear mixed model fit by REML. t-tests use Satterthwaite's method [
lmerModLmerTest]
Formula: ER ~ EPOCH * CTX + (1 | ID) + (1 | WORD)
   Data: dd

REML criterion at convergence: -949.5

Scaled residuals:
    Min      1Q  Median      3Q     Max
-2.8468 -0.4893 -0.1287  0.2928  5.3315

Random effects:
 Groups   Name        Variance Std.Dev.
 WORD     (Intercept) 0.002836 0.05325
 ID       (Intercept) 0.001605 0.04006
 Residual             0.017892 0.13376
Number of obs: 906, groups:  WORD, 39; ID, 9

Fixed effects:
                     Estimate Std. Error         df t value Pr(>|t|)
(Intercept)         1.248e-03  2.597e-02  5.552e+01   0.048 0.961835
EPOCHACC1          -2.083e-04  2.115e-02  8.530e+02  -0.010 0.992143
EPOCHACC2           9.722e-03  2.115e-02  8.530e+02   0.460 0.645857
CTXONSET            6.488e-02  3.037e-02  7.179e+01   2.137 0.036040 *
CTXCODA             8.587e-02  2.859e-02  7.248e+01   3.003 0.003664 **
EPOCHACC1:CTXONSET  2.346e-03  2.871e-02  8.530e+02   0.082 0.934877
EPOCHACC2:CTXONSET  1.091e-01  2.871e-02  8.530e+02   3.799 0.000155 ***
EPOCHACC1:CTXCODA   5.629e-02  2.700e-02  8.530e+02   2.085 0.037396 *
EPOCHACC2:CTXCODA   2.639e-01  2.700e-02  8.530e+02   9.773  < 2e-16 ***
---
Signif. codes:  0 '***' 0.001 '**' 0.01 '*' 0.05 '.' 0.1 ' ' 1

            Class   Family     Link  Marginal Conditional       AIC
1 lmerModLmerTest gaussian identity 0.3666533   0.4925932 -980.8992
```

Model M2: log(MVT)

Predict log(MVT) from fixed effects of epoch and error, including random slopes for error by speaker and random intercepts by word pair

```
Linear mixed model fit by REML. t-tests use Satterthwaite's method [
lmerModLmerTest]
Formula: MVT ~ EPOCH + ERROR + (ERROR | ID) + (1 | WORD)
   Data: d

REML criterion at convergence: 789.5

Scaled residuals:
    Min      1Q  Median      3Q     Max
-3.5805 -0.6145 -0.0061  0.5691  3.6029

Random effects:
 Groups   Name        Variance Std.Dev. Corr
 WORD     (Intercept) 0.01396  0.1181
 ID       (Intercept) 0.10585  0.3253
          ERRORTRUE   0.02038  0.1428   -0.16
 Residual             0.12332  0.3512
Number of obs: 906, groups:  WORD, 39; ID, 9

Fixed effects:
             Estimate Std. Error        df t value Pr(>|t|)
(Intercept)   2.09924    0.11227   8.81013  18.698 2.15e-08 ***
EPOCHACC1     0.06207    0.02882 852.68607   2.153   0.0316 *
EPOCHACC2     0.17602    0.03049 871.06325   5.774 1.08e-08 ***
ERRORTRUE     0.11354    0.05624   9.91043   2.019   0.0714 .
---

            Class   Family     Link   Marginal Conditional      AIC
1 lmerModLmerTest gaussian identity 0.04266834   0.5193071 790.1511
```

Model M3: log(VEL) by epoch

Predict log(VEL) from fixed effects of epoch and error, including random slopes for error by speaker and random intercepts by word pair

```
Linear mixed model fit by REML. t-tests use Satterthwaite's method [
lmerModLmerTest]
Formula: VEL ~ EPOCH + ERROR + (ERROR | ID) + (1 | WORD)
   Data: d

REML criterion at convergence: 865.4

Scaled residuals:
    Min      1Q  Median      3Q     Max
-3.1343 -0.6007 -0.0245  0.5953  4.2750

Random effects:
 Groups   Name        Variance Std.Dev. Corr
 WORD     (Intercept) 0.01357  0.1165
 ID       (Intercept) 0.12326  0.3511
          ERRORTRUE   0.02287  0.1512   -0.49
 Residual             0.13476  0.3671
Number of obs: 906, groups:  WORD, 39; ID, 9

Fixed effects:
             Estimate Std. Error        df t value Pr(>|t|)
(Intercept)   1.58799    0.12072   8.70279  13.155 4.84e-07 ***
EPOCHACC1     0.14419    0.03013 853.54712   4.786 2.01e-06 ***
EPOCHACC2     0.39037    0.03185 872.61164  12.257  < 2e-16 ***
ERRORTRUE     0.12920    0.05924   9.40875   2.181   0.0558 .
---

            Class   Family     Link  Marginal Conditional     AIC
1 lmerModLmerTest gaussian identity 0.1228869   0.5438895 866.236

 contrast      estimate     SE  df t.ratio p.value
 STABLE - ACC1   -0.144 0.0301 854  -4.786 <.0001
 STABLE - ACC2   -0.390 0.0318 873 -12.257 <.0001
 ACC1 - ACC2     -0.246 0.0308 862  -7.983 <.0001

Results are averaged over the levels of: ERROR
Degrees-of-freedom method: satterthwaite
P value adjustment: tukey method for comparing a family of 3 estimates
```

Model M4: log(VEL) by local error

Predict error-local log(VEL) with fixed effects of epoch, context and PRE/POST, with random intercepts by speaker and word pair

```
Linear mixed model fit by REML. t-tests use Satterthwaite's method [
lmerModLmerTest]
Formula: VEL ~ EPOCH + CTX + PP + (1 | ID)
   Data: e

REML criterion at convergence: 2450.6

Scaled residuals: 
    Min      1Q  Median      3Q     Max 
-4.5742 -0.6222 -0.0149  0.6638  4.8257 

Random effects:
 Groups   Name        Variance Std.Dev.
 ID       (Intercept) 0.1164   0.3412  
 Residual             0.1731   0.4160  
Number of obs: 2198, groups:  ID, 9

Fixed effects:
              Estimate Std. Error         df t value Pr(>|t|)    
(Intercept)    0.40424    0.13806   16.88551   2.928 0.009440 ** 
EPOCHACC1     -0.01644    0.02912 2184.33052  -0.565 0.572424    
EPOCHACC2      0.07425    0.02521 2184.67584   2.945 0.003267 ** 
CTXONSET       0.18511    0.07653 2184.74600   2.419 0.015652 *  
CTXCODA        0.22124    0.07557 2184.90474   2.928 0.003449 ** 
PPPOST         0.05916    0.01775 2183.92526   3.333 0.000872 ***
---

            Class   Family     Link   Marginal Conditional      AIC
1 lmerModLmerTest gaussian identity 0.01170737   0.4091666 2437.073
```

Model M5: log(VEL) by local error (test error type)

subset: excludes substitutions, non-alternating controls

Predict error-local log(VEL) from fixed effects of context, error type, articulator and PRE/POST, with random slopes for context and type by speaker, and random intercepts by word pair; includes interactions between context and error type, and between context and articulator

```
Linear mixed model fit by REML. t-tests use Satterthwaite's method [
lmerModLmerTest]
Formula: VEL ~ PP + CTX + TYPE + ART + CTX:TYPE + CTX:ART + (CTX * TYPE |
    ID) + (1 | WORD)
   Data: ee
Control: lmerControl(optimizer = "bobyqa", optCtrl = list(maxfun = 2e+06))

REML criterion at convergence: 2006.6

Scaled residuals:
    Min      1Q  Median      3Q     Max
-3.6817 -0.6080  0.0115  0.6869  3.7626

Random effects:
 Groups   Name            Variance Std.Dev. Corr
 WORD     (Intercept)     0.02202  0.1484
 ID       (Intercept)     0.08454  0.2908
          CTXCODA         0.03894  0.1973    0.09
          TYPERED         0.05257  0.2293    0.10  0.10
          CTXCODA:TYPERED 0.07521  0.2742   -0.37 -0.18 -0.94
 Residual                 0.14497  0.3807
Number of obs: 2028, groups:  WORD, 28; ID, 9

Fixed effects:
                  Estimate Std. Error         df t value Pr(>|t|)
(Intercept)        0.47118    0.12065   17.20932   3.906  0.00112 **
PPPOST             0.05678    0.01691 1958.88955   3.358  0.00080 ***
CTXCODA            0.21044    0.10643   28.49435   1.977  0.05776 .
TYPERED            0.32331    0.10618    5.95954   3.045  0.02286 *
ARTTD              0.16925    0.06164 1393.25749   2.746  0.00612 **
ARTTT              0.16105    0.06471 1458.77351   2.489  0.01293 *
CTXCODA:TYPERED   -0.32235    0.12018    4.83074  -2.682  0.04527 *
CTXCODA:ARTTD     -0.18653    0.06820 1549.53476  -2.735  0.00631 **
CTXCODA:ARTTT     -0.22351    0.07196 1590.17623  -3.106  0.00193 **
---

            Class   Family     Link   Marginal Conditional      AIC
1 lmerModLmerTest gaussian identity 0.01707547   0.4864858 2009.397
```

```
CTX = ONSET:
 contrast  estimate    SE   df t.ratio p.value
 INT - RED -0.32331 0.106 5.96 -3.045  0.0229

CTX = CODA:
 contrast  estimate    SE   df t.ratio p.value
 INT - RED -0.00096 0.040 4.32 -0.024  0.9819

Results are averaged over the levels of: PP, ART
Degrees-of-freedom method: satterthwaite

CTX = ONSET:
 contrast estimate     SE   df t.ratio p.value
 LA - TD   -0.1692 0.0616 1393 -2.746  0.0168
 LA - TT   -0.1610 0.0647 1459 -2.489  0.0345
 TD - TT    0.0082 0.0383 1787  0.214  0.9750

CTX = CODA:
 contrast estimate     SE   df t.ratio p.value
 LA - TD    0.0173 0.0294 1628  0.589  0.8262
 LA - TT    0.0625 0.0317 1943  1.969  0.1201
 TD - TT    0.0452 0.0307 1299  1.470  0.3059

Results are averaged over the levels of: PP, TYPE
Degrees-of-freedom method: satterthwaite
P value adjustment: tukey method for comparing a family of 3 estimates
```

Model M6: AMI

subset: excludes non-alternating controls
maps MIH1,MIH2A,MIH2B,MIHJ to MI coded by PAIR (encodes the HEAD-paired articulator)

Predict MI from fixed effects of epoch, context, and PAIR with random intercepts by speaker and word pair; interaction terms for epoch:context and context:PAIR

```
Linear mixed model fit by REML. t-tests use Satterthwaite's method Formula:
MI ~ EPOCH + CTX + PAIR + EPOCH:CTX + CTX:PAIR + (1 | ID) + (1 |
    WORD)
   Data: dd

REML criterion at convergence: -4164.7

Scaled residuals:
    Min      1Q  Median      3Q     Max
-3.2068 -0.6705 -0.0692  0.5783  5.1086

Random effects:
 Groups   Name        Variance  Std.Dev.
 WORD     (Intercept) 0.0009765 0.03125
 ID       (Intercept) 0.0176400 0.13282
 Residual             0.0173890 0.13187
Number of obs: 3624, groups:  WORD, 39; ID, 9

Fixed effects:
                     Estimate Std. Error         df t value Pr(>|t|)
(Intercept)         7.789e-01  4.649e-02  9.672e+00  16.754 1.82e-08 ***
EPOCHACC1           3.725e-02  1.043e-02  3.563e+03   3.573 0.000357 ***
EPOCHACC2           8.770e-03  1.043e-02  3.563e+03   0.841 0.400260
CTXONSET            3.118e-02  1.936e-02  1.188e+02   1.611 0.109896
CTXCODA             1.153e-02  1.822e-02  1.199e+02   0.633 0.527949
PAIRMIH2A           9.405e-03  1.204e-02  3.563e+03   0.781 0.434701
PAIRMIH2B           9.405e-03  1.204e-02  3.563e+03   0.781 0.434701
PAIRMIHJ            6.923e-02  1.204e-02  3.563e+03   5.751 9.61e-09 ***
EPOCHACC1:CTXONSET -3.096e-02  1.415e-02  3.563e+03  -2.188 0.028741 *
EPOCHACC2:CTXONSET  2.583e-02  1.415e-02  3.563e+03   1.825 0.068033 .
EPOCHACC1:CTXCODA   2.475e-03  1.331e-02  3.563e+03   0.186 0.852489
EPOCHACC2:CTXCODA   6.511e-02  1.331e-02  3.563e+03   4.892 1.04e-06 ***
CTXONSET:PAIRMIH2A  8.192e-02  1.634e-02  3.563e+03   5.014 5.59e-07 ***
CTXCODA:PAIRMIH2A   7.912e-02  1.537e-02  3.563e+03   5.148 2.77e-07 ***
CTXONSET:PAIRMIH2B  9.154e-02  1.634e-02  3.563e+03   5.603 2.27e-08 ***
CTXCODA:PAIRMIH2B   8.545e-02  1.537e-02  3.563e+03   5.560 2.89e-08 ***
CTXONSET:PAIRMIHJ   1.178e-02  1.634e-02  3.563e+03   0.721 0.470851
CTXCODA:PAIRMIHJ   -2.838e-02  1.537e-02  3.563e+03  -1.847 0.064890 .
---

            Class   Family     Link   Marginal Conditional       AIC
1 lmerModLmerTest gaussian identity 0.07346708   0.5525261 -4253.907
```

```
CTX = SAME:
 contrast      estimate      SE   df z.ratio p.value
 MIH1 - MIH2A  -0.00940 0.01204 3563 -0.781  0.8630
 MIH1 - MIH2B  -0.00940 0.01204 3563 -0.781  0.8630
 MIH1 - MIHJ   -0.06923 0.01204 3563 -5.751  <.0001
 MIH2A - MIH2B  0.00000 0.01204 3563  0.000  1.0000
 MIH2A - MIHJ  -0.05983 0.01204 3563 -4.970  <.0001
 MIH2B - MIHJ  -0.05983 0.01204 3563 -4.970  <.0001

CTX = ONSET:
 contrast      estimate      SE   df z.ratio p.value
 MIH1 - MIH2A  -0.09133 0.01105 3563 -8.267  <.0001
 MIH1 - MIH2B  -0.10094 0.01105 3563 -9.138  <.0001
 MIH1 - MIHJ   -0.08101 0.01105 3563 -7.334  <.0001
 MIH2A - MIH2B -0.00961 0.01105 3563 -0.870  0.8203
 MIH2A - MIHJ   0.01031 0.01105 3563  0.934  0.7867
 MIH2B - MIHJ   0.01993 0.01105 3563  1.804  0.2714

CTX = CODA:
 contrast      estimate      SE   df z.ratio p.value
 MIH1 - MIH2A  -0.08852 0.00955 3563 -9.266  <.0001
 MIH1 - MIH2B  -0.09486 0.00955 3563 -9.928  <.0001
 MIH1 - MIHJ   -0.04085 0.00955 3563 -4.276  0.0001
 MIH2A - MIH2B -0.00633 0.00955 3563 -0.663  0.9111
 MIH2A - MIHJ   0.04767 0.00955 3563  4.990  <.0001
 MIH2B - MIHJ   0.05401 0.00955 3563  5.653  <.0001

Results are averaged over the levels of: EPOCH
Degrees-of-freedom method: asymptotic
P value adjustment: tukey method for comparing a family of 4 estimates
```

Model M7: MP (excludes controls, includes errors)

subset: excludes non-alternating controls
maps MPH1,MPH2A,MPH2B to PAIR (encodes the HEAD-paired articulator)

Predict MP from fixed effects of epoch, context, error, and PAIR; interaction term between error and context, random intercepts by speaker, and random slopes for pair by word

```
Linear mixed model fit by REML. t-tests use Satterthwaite's method [
lmerModLmerTest]
Formula: MP ~ EPOCH + CTX + ERROR + PAIR + CTX:ERROR + (1 | ID) + (PAIR |
    WORD)
   Data: dd
Control: lmerControl(optimizer = "bobyqa", optCtrl = list(maxfun = 2e+05))

REML criterion at convergence: 11211.4

Scaled residuals:
    Min      1Q  Median      3Q     Max
-4.5028 -0.6572  0.0470  0.6906  2.9439

Random effects:
 Groups   Name        Variance Std.Dev. Corr
 WORD     (Intercept)  5.342   2.311
          PAIRMPH2A2   6.423   2.534    -0.88
          PAIRMPH2B2   6.695   2.587    -0.84  0.83
 ID       (Intercept)  4.943   2.223
 Residual             14.748   3.840
Number of obs: 1998, groups:  WORD, 28; ID, 9

Fixed effects:
                Estimate Std. Error        df t value Pr(>|t|)
(Intercept)      16.9014     0.9217   17.6892  18.338 5.93e-13 ***
EPOCHACC1        -0.1534     0.2132 1902.7494  -0.720    0.472
EPOCHACC2        -1.5520     0.2319 1914.1063  -6.693 2.87e-11 ***
CTXCODA           0.3176     0.4675   38.9177   0.679    0.501
ERROR+            0.8928     0.3004 1864.8055   2.972    0.003 **
PAIRMPH2A2        4.4536     0.5249   26.9599   8.485 4.31e-09 ***
PAIRMPH2B2        4.5339     0.5341   26.4323   8.489 5.00e-09 ***
CTXCODA:ERROR+   -0.9639     0.3740 1915.6365  -2.577    0.010 *
---

            Class   Family     Link  Marginal Conditional      AIC
1 lmerModLmerTest gaussian identity 0.1806585   0.4650181 11240.68
```

Model M8: MP (includes controls, excludes errors)

subset: excludes epochs with errors
maps MPH11,MPH12,MPH2A1,MPH2A2,MPH2B1,MPH2B2,MPHJ1,MPHJ2 to PAIR (encodes the HEAD-paired articulator)

Predict MP from fixed effects of epoch, context, and pair, with random intercepts by speaker and word pair; interaction terms for epoch:context and context:PAIR

```
Linear mixed model fit by REML. t-tests use Satterthwaite's method [
lmerModLmerTest]
Formula: MP ~ EPOCH + CTX + PAIR + EPOCH:CTX + CTX:PAIR + (1 | ID) + (1 |
    WORD)
   Data: dd

REML criterion at convergence: 24203.4

Scaled residuals:
    Min      1Q  Median      3Q     Max
-4.1673 -0.6404  0.0269  0.6434  3.0227

Random effects:
 Groups   Name        Variance Std.Dev.
 WORD     (Intercept)  2.286   1.512
 ID       (Intercept)  5.722   2.392
 Residual             18.757   4.331
Number of obs: 4176, groups:  WORD, 39; ID, 9

Fixed effects:
                     Estimate Std. Error        df t value Pr(>|t|)
(Intercept)           17.8805     0.9739   17.0454  18.360 1.15e-12 ***
EPOCHACC1             -0.7812     0.2455 4103.4019  -3.182 0.001473 **
EPOCHACC2             -1.9126     0.2498 4103.6911  -7.656 2.38e-14 ***
CTXONSET              -1.0290     0.8064   76.3611  -1.276 0.205816
CTXCODA               -0.7086     0.7821   86.7544  -0.906 0.367430
PAIRMPH12             -1.3421     0.4047 4102.4955  -3.316 0.000921 ***
PAIRMPH2A1            -0.7877     0.4047 4102.4955  -1.946 0.051698 .
PAIRMPH2A2            -1.9875     0.4047 4102.4955  -4.911 9.44e-07 ***
PAIRMPH2B1            -0.7877     0.4047 4102.4955  -1.946 0.051698 .
PAIRMPH2B2            -1.9875     0.4047 4102.4955  -4.911 9.44e-07 ***
PAIRMPHJ1             -3.6694     0.4047 4102.4955  -9.066  < 2e-16 ***
PAIRMPHJ2             -3.8431     0.4047 4102.4955  -9.495  < 2e-16 ***
EPOCHACC1:CTXONSET     0.8140     0.3689 4117.6932   2.206 0.027412 *
EPOCHACC2:CTXONSET     0.3038     0.4376 4121.1462   0.694 0.487549
EPOCHACC1:CTXCODA      0.6608     0.3750 4120.5769   1.762 0.078138 .
EPOCHACC2:CTXCODA      1.4803     0.5415 4135.2602   2.734 0.006291 **
CTXONSET:PAIRMPH12     2.1617     0.6371 4102.4955   3.393 0.000697 ***
CTXCODA:PAIRMPH12      0.6771     0.6600 4102.4955   1.026 0.305023
CTXONSET:PAIRMPH2A1    0.2887     0.6371 4102.4955   0.453 0.650449
```

```
CTXCODA:PAIRMPH2A1     0.4356     0.6600 4102.4955   0.660 0.509344
CTXONSET:PAIRMPH2A2    6.5185     0.6371 4102.4955  10.232  < 2e-16 ***
CTXCODA:PAIRMPH2A2     5.8473     0.6600 4102.4955   8.859  < 2e-16 ***
CTXONSET:PAIRMPH2B1    0.7603     0.6371 4102.4955   1.193 0.232784
CTXCODA:PAIRMPH2B1     0.6780     0.6600 4102.4955   1.027 0.304420
CTXONSET:PAIRMPH2B2    6.5511     0.6371 4102.4955  10.283  < 2e-16 ***
CTXCODA:PAIRMPH2B2     6.4077     0.6600 4102.4955   9.708  < 2e-16 ***
CTXONSET:PAIRMPHJ1     0.3345     0.6371 4102.4955   0.525 0.599538
CTXCODA:PAIRMPHJ1      0.7553     0.6600 4102.4955   1.144 0.252563
CTXONSET:PAIRMPHJ2     2.9701     0.6371 4102.4955   4.662 3.23e-06 ***
CTXCODA:PAIRMPHJ2      1.3059     0.6600 4102.4955   1.979 0.047936 *
---

            Class   Family     Link  Marginal Conditional      AIC
1 lmerModLmerTest gaussian identity 0.1750878   0.4218783 24267.18
```
